# Stabilizing breast implants with the double anchor capsule flap technique: insights from a retrospective case series of 30 patients

**DOI:** 10.1016/j.jpra.2026.10.018

**Published:** 2026-10-25

**Authors:** Diego Armando Franco Arias, Christopher Michael Edwards, Lucas Tomkowski Cancian, Carlos Andres Valverde Solano

**Affiliations:** a Interquirofanos, Cl. 7 #39 107 Piso3 y 4, Medellín, Colombia; b Pontifícia Universidade Católica do Rio Grande do Sul: Porto Alegre, Rio Grande do Sul, BR, Av. Ipiranga, 6681 - Partenon, Porto Alegre - RS, 90619-900, Brasil; c COSMOS SCIENTIFIC, INC, 20803 SW 83rd Ave, Cutler Bay, FL, 33189, United States

**Keywords:** Breast, Implant, Stabilization, Technique, Secondary mastopexy, Ptosis

## Abstract

**Background:**

Breast deformities such as ptosis, bottoming out, atrophy, sagging, and implant malposition are common challenges following breast augmentation or augmentation-mastopexy procedures. Multiple factors—including implant volume, placement plane, surgical technique, and gravitational or muscular forces—affect long-term implant stability.

**Objective:**

To describe the Double-Anchor Capsule Flap Technique (DAT) for breast remodeling in revision mastopexy and evaluate its safety and effectiveness in a retrospective series.

**Methods:**

This retrospective case series included 30 bilateral cases performed between October 2023 and September 2024. Inclusion criteria were prior breast augmentation, ptosis of any degree, BMI 20–35, adequate flap thickness (≥3 cm), and suitability for Wise pattern skin resection. Exclusion criteria included prior liposuction in axillary/intercostal regions, ASIA or BII, restrictive illnesses, smoking, and reconstructive surgery within 12 months. Data collected included patient demographics, implant characteristics, surgical technique, intraoperative flap viability, complications, and outcomes at one-year follow-up.

**Results:**

Flap survival was achieved in all patients. At one-year follow-up, all patients demonstrated stable breast shape, optimal nipple-areola complex positioning, and implant projection. No recurrent ptosis, implant malposition, or BIA-ALCL was observed. No postoperative complications were reported during the 12-month follow-up period. Average implant volume was reduced by 22.3 cc (SD 25.9 cc) to minimize eccentric forces, and dual anchor lines provided reliable long-term implant support.

**Conclusion:**

The Double-Anchor Capsule Flap Technique is a safe and effective approach for stabilizing breast implants and correcting post-augmentation deformities. By preserving vascularized capsular tissue and providing dual support, DAT enhances long-term stability and aesthetic outcomes while minimizing the use of foreign materials. Careful patient selection and precise surgical planning are key to optimal results.

## Introduction

Breast deformities such as ptosis, bottoming out, atrophy, sagging, and double-bubble deformity after augmentation or augmentation-mastopexy remain a frequent challenge in plastic surgery. Implant malposition occurs in 1.5–2.7% of augmentation cases, and surgical correction or adjustment is required in 5.9% of smooth round implants and 1.4% of textured anatomical implants.^1^^,^^2^

Various authors have described capsular flaps and capsulorrhaphy to correct implant malposition and reinforce the lower pole, including Carlsen’s vascularized capsular sling for implant repositioning and Gargano’s multilayered capsular flap technique for implant exposure salvage.

Unlike prior techniques, DAT preserves the vascularized capsular architecture and creates a full-thickness inferior external capsular flap that provides dynamic inferior-pole support. The flap helps reposition the implant medially and superiorly, counteracting the typical lateral and inferior displacement caused by pectoralis major muscle activity.

This technique differs from the capsular hammock described by Wessels et al. in that it focuses on implant stabilization and pocket tightening without repositioning the inframammary fold, while redefining the pocket’s base, shape, and dimensions.^3^

Unlike simple capsular plication, isolated capsulorrhaphy sutures, or nonautologous internal support materials, our method uses excess lapel-like capsule tissue advanced and secured with two parallel fixation suture lines. This double-layer fixation provides redundancy and maintains stability even if one suture line fails.

Various authors have described capsular flap and capsulorrhaphy techniques to correct implant malposition and reinforce the lower pole. The Double-Anchor Capsule Flap Technique (DAT) differs by preserving the vascularized capsule and creating a full-thickness inferolateral flap that provides dual support against lateral and inferior implant displacement, without using foreign materials or requiring inframammary fold repositioning.

The criteria for capsulectomy during revision mastopexy remain unclear. Accepted indications include encapsulation, chronic capsulitis (biopsy-confirmed), implant rupture with capsule infiltration, and calcification. Imaging-detected pathological capsule changes may also justify partial or total capsulectomy.^4^ Indications are increasingly influenced by Breast Implant Illness or ASIA,^4^ which may be paradoxical when implant replacement is planned. Total capsulectomy can weaken surrounding tissues and expose patients to an uncertain healing environment regarding implant stability.

Polyurethane prostheses increase implant stability through greater adhesion but carry risks, including Breast Implant-Associated Anaplastic Large Cell Lymphoma (BIA-ALCL).^5^^,^^6^ The frustration for both patients and surgeons in these cases highlights the need to master multiple techniques for safe correction of implant ptosis–related deformities.

In this case series, we describe an innovative capsular flap technique for implant stabilization in secondary mastopexies as an alternative approach to prevent and correct ptosis and posterior atrophy.

## Objectives

This case series describes the Double-Anchor Capsule Flap Technique (DAT) for breast remodeling in revision mastopexy after prior augmentation and evaluates its safety, effectiveness, and advantages over conventional corrective methods.

## Patients and methods

***Study design.*** This retrospective case series includes 30 bilateral cases in which the Double-Anchor Technique was used for breast remodeling during revision mastopexy.

***Setting:*** All procedures were performed between October 2023 and September 2024 at a single plastic surgery center in Medellín, Colombia.

***Patient selection.*** *Inclusion criteria:* a) Prior breast augmentation surgery scheduled for breast remodeling during revision mastopexy surgery; b) Mammary gland ptosis; c) In the opinion of the surgeon, the application of the WISE technique^7^ is clinically appropriate; d) Body Mass Index (BMI) between 20 and 35; e) Pinch test in the flap area greater than or equal to 3 cm in thickness. *Exclusion criteria:* a) Previous liposuction procedures in the axillary and intercostal regions; b) Autoimmune/Autoinflammatory Syndrome Induced by Adjuvants (ASIA) or Breast Implant Illness (BII); c) Restrictive pathologies or illnesses; d) Smokers; e) Reconstructive surgeries within 365 days prior to surgery.

***Outcomes:*** Primary outcomes were flap viability, implant stability, and postoperative complications. Secondary outcomes included breast shape, nipple–areola complex position, and implant projection. Potential confounders such as BMI, implant size, and prior surgical history were also recorded.

***Data collection.*** The study included consecutive eligible patients undergoing breast remodeling with the Double-Anchor Capsule Flap Technique (DAT) during revision mastopexy for deformities following prior augmentation. Demographics, implant characteristics, surgical details, and preoperative imaging were systematically collected using standardized instruments. All data were anonymized to ensure confidentiality.

***Data sources and measurement.*** Data were obtained from medical records and preoperative imaging. Demographics were extracted from operative notes. Surgical outcomes were assessed clinically at each follow-up, with standardized measurements to ensure consistency across patients.

***Bias:*** This retrospective case series may be subject to selection and information bias. To reduce this, all consecutive eligible patients were included, surgeries were performed by a single experienced surgeon, and data were collected using standardized forms. Follow-up was uniform (12 months), and outcomes were recorded consistently.

***Sample size:*** No formal sample size calculation was performed due to the retrospective case series design.

***Quantitative variables:*** Patient age, BMI, implant volume, and flap thickness were summarized using means and ranges. Continuous variables were analyzed as continuous measures, recorded in standard units to ensure consistency across the cohort.

***Statistical methods:*** *Data were analyzed descriptively using means and ranges. No inferential tests, subgroup analyses, or confounder adjustments were performed due to the single-group design. There were no missing outcome data, and all patients completed 12-month follow-up; no sensitivity analyses were conducted.*

## Procedure: double-anchor capsule flap technique

All patients received prophylactic intravenous antibiotics and were positioned supine under sterile conditions.

### Anatomy and preoperative landmarks

Accurate preoperative marking is essential for successful Wise pattern mastopexy in secondary revision after prior augmentation or mastopexy. The Wise pattern (inverted-T incision) enables precise reshaping and repositioning of breast tissue and skin for optimal aesthetic outcomes.^7^

### Patient positioning

The patient is placed in an upright standing position with arms at the sides to allow natural breast drape and accurate assessment of breast anatomy and skin excess.

### Anatomical landmarks identification

*Midline Reference: A* vertical line is drawn from the suprasternal notch to the umbilicus to establish the body midline.

*Breast Meridian:* A vertical line is extended from the midclavicular point through the nipple to the inframammary fold (IMF), defining the breast meridian.

*Inframammary Fold (IMF):* The existing IMF is palpated and marked bilaterally. In cases of ptosis or implant displacement, a higher desired IMF may be marked to account for tissue resection and lifting.

*Nipple-Areola Complex (NAC) Position:* The ideal NAC position is determined, typically at the level of the inframammary fold when measured from the suprasternal notch (about 19–21 cm depending on height and torso length). A point is marked for the new breast apex and NAC center.

### Wise pattern marking

*Keyhole Design:* A keyhole (pedicle) design is drawn with two curved lines from the new NAC position. The superior limb width corresponds to the desired areolar diameter (typically 4–5 cm).

*Vertical Limbs:* From the inferior keyhole points, vertical lines are drawn toward the IMF to define the vertical incision. Their length depends on skin excess and required lift.^8^

*Inframammary Incision Line:* A horizontal line is drawn along the IMF connecting the inferior ends of the vertical limbs. Its length is adjusted based on tissue resection needs and the extent of lateral or medial skin removal.

### Adjustments for secondary revisions

*Assessment of Previous Scars and Implants:* Existing scars are evaluated and markings adjusted to incorporate or revise them as needed. The position and condition of existing implants are assessed to plan for replacement or adjustment.

*Skin Quality and Tissue Assessment:* Skin elasticity, thickness, and areas of thinning or prior damage are identified. Markings may be modified to preserve adequate blood supply to the nipple-areola complex (NAC) and surrounding tissues.

### Intraoperative considerations

*Verification in Different Positions:* After initial markings, the patient may be asked to raise arms or lean slightly forward to assess changes in breast shape and adjust markings accordingly.

*Symmetry Checks:* Measurements are compared between both breasts to ensure symmetry in NAC position, incision lines, and overall breast shape.

*Consent Confirmation:* The surgical plan and markings are reviewed with the patient to confirm understanding and consent.

### Intraoperative marking

The flap design is marked in the standing position prior to surgery. Incisions are then made according to the preoperative plan with intraoperative adjustments as needed.

Accurate identification of anatomical structures and preoperative landmarks is essential for the successful application of the Double-Anchor Capsule Flap Technique (DAT). After exposure of the breast capsule, its condition is assessed using the Baker classification for capsular contracture.^9^ In Baker I–II cases, indicating minimal to mild contracture, the procedure proceeds to marking prior to implant explantation.

During this phase, anchor placement sites are marked on the capsule. These markings guide proper implant support and positioning postoperatively, corresponding to anatomical landmarks and adjusted to the patient’s breast anatomy and degree of ptosis.

The marking process involves:

*Identifying Key Anatomical Points:* The inframammary fold, lateral and medial borders of the breast, and the apex of the breast mound are identified.

*Determining Anchor Placement Sites:* Based on assessment, the surgeon marks superior and medial capsule anchor points to optimize support and symmetry.

*Ensuring Symmetry:* Bilateral markings are compared to ensure symmetrical anchor placement, essential for achieving aesthetically balanced results.

By establishing these landmarks before implant removal, the surgeon maintains spatial orientation and improves precision. This facilitates accurate execution of the DAT, enhancing implant stabilization and reducing the risk of postoperative ptosis and atrophy.

If the capsule is graded higher than Baker II, indicating significant contracture, capsulectomy or capsulotomy may be required. For Baker I–II capsules, the marking technique allows effective execution of the DAT and favorable surgical outcomes.

***De-epithelization.*** De-epithelialization is performed using the Schwartzman maneuver in an inverted Wise pattern, followed by resection of the inferior dermoglandular flap to expose the capsule containing the implant. The NAC pedicle is designed as superior or superomedial depending on NAC position and degree of ptosis.^8^

***Flap Design:*** Using a marker or methylene blue, the inferolateral capsule flap is drawn at a 90° angle oriented medially and superiorly. This flap is then mobilized and fixed in medial and superior vectors to correct implant malposition, typically characterized by inferior and lateral displacement due to gravity and pectoralis major activity. Wider markings allow greater flap mobilization and stronger fixation (Fig. 1, Fig. 4).

**Fig. 1:**
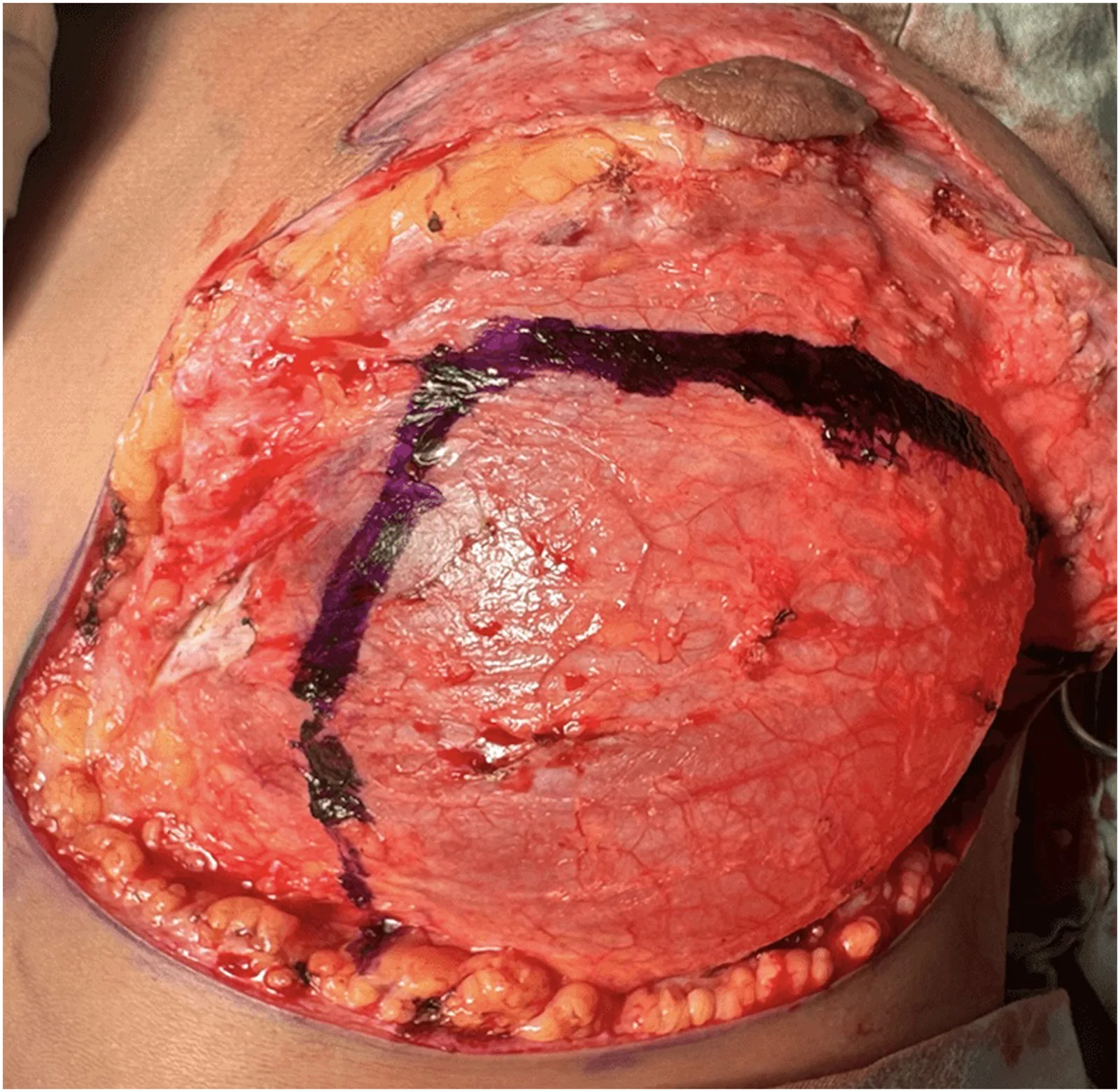
Surgical procedure - A. Flap design. A flap is outlined in the inferolateral quadrant at a precise 90-degree angle, oriented using the medial and superior vectors. **Case 1: Description:** Surgical procedure.

***Flap Dissection:*** The redundant dermoglandular flap is resected using electrocautery, starting at the inframammary fold. Careful dissection preserves as much physiological capsule as possible. This exposes the lower pole of the intact capsule containing the prosthesis and allows better assessment of the flap design.

Once the capsule is incised, the triangular flap is mobilized medially and superiorly and overlapped as needed at the surgeon’s discretion (Fig. 2, Fig. 5). The first anchor line is placed with non-absorbable sutures along the medial capsular free edge, and the second along the medial capsular insertion. This double-anchor system provides prosthesis stability, ensuring that if one line fails, the other maintains support (Fig. 3, Videos 1–2).

**Fig. 2:**
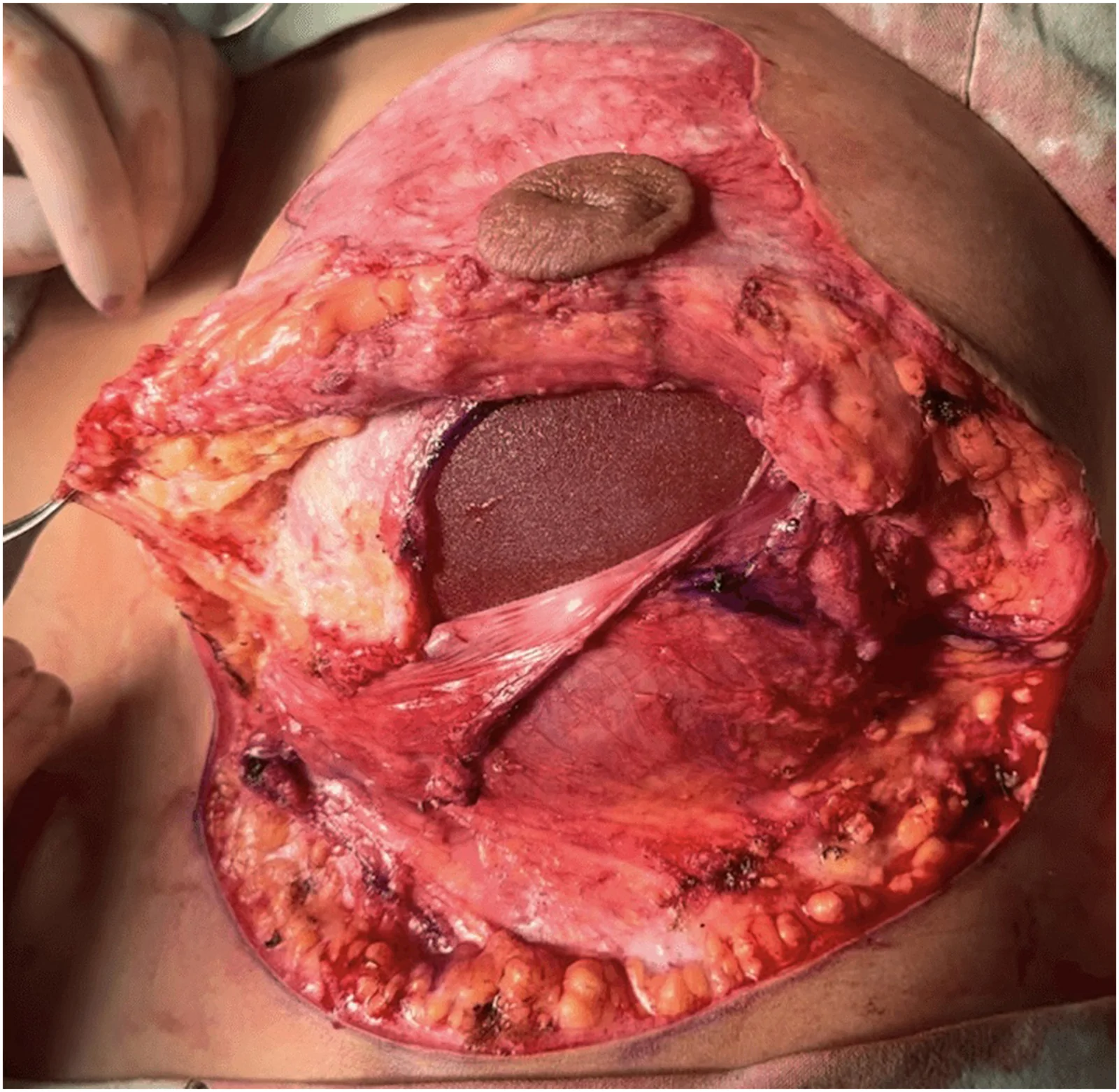
Surgical procedure - B. Flap advancement. The flap is advanced in a superomedial manner and anchored at the angle so that the implant placement is assessed.

**Fig. 3:**
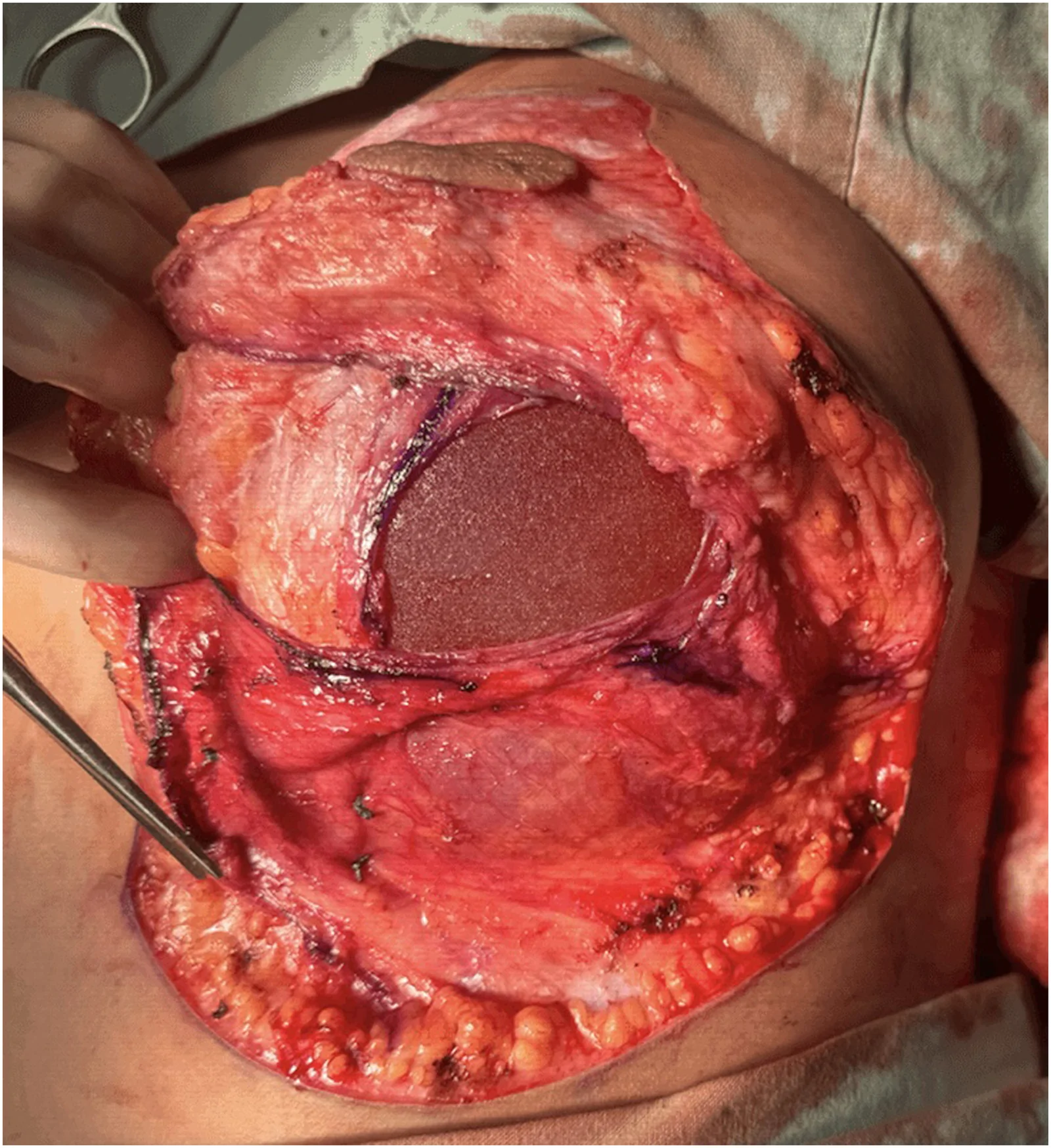
Surgical procedure - C. Flap anchorage. When the two lines of anchoring are secured with non-absorbable sutures, the position of the implant is evaluated and the need of closing the superior vector of the flap is performed.

Surgeons must avoid excessive tension when closing the first anchoring line, as this may cause implant folding or distortion. If this occurs, the anchoring line should be adjusted to ensure proper implant positioning.

Inferior-lateral malposition is often associated with hyperactivity of hypertrophic muscle bundles in dual-plane pocket designs. To mitigate this, the surgeon may consider strategies such as inverted dual-plane modification, partial or total myotomy, or selective muscle weakening.

***Hemostasis and Viability.*** Strict hemostatic control was maintained throughout the procedure. Because the capsular flap was completely buried and de-epithelialized, conventional skin flap monitoring parameters such as color, tissue turgor, and capillary refill were not applicable. Intraoperative viability was ensured by preserving pedicle continuity and avoiding excessive tension, pedicle kinking, or compression during flap transposition and fixation.

Indocyanine green (ICG) angiography was not used in this retrospective series. Flap perfusion was assessed using intraoperative clinical judgment and serial postoperative examinations.

***Closure.*** Closure of the glandular pillars, elevation of the nipple-areola complex, and skin closure are performed at the surgeon's discretion (Video 3). A video animation explaining the full procedure is included in Video 4 Fig. 6.

**Fig. 4:**
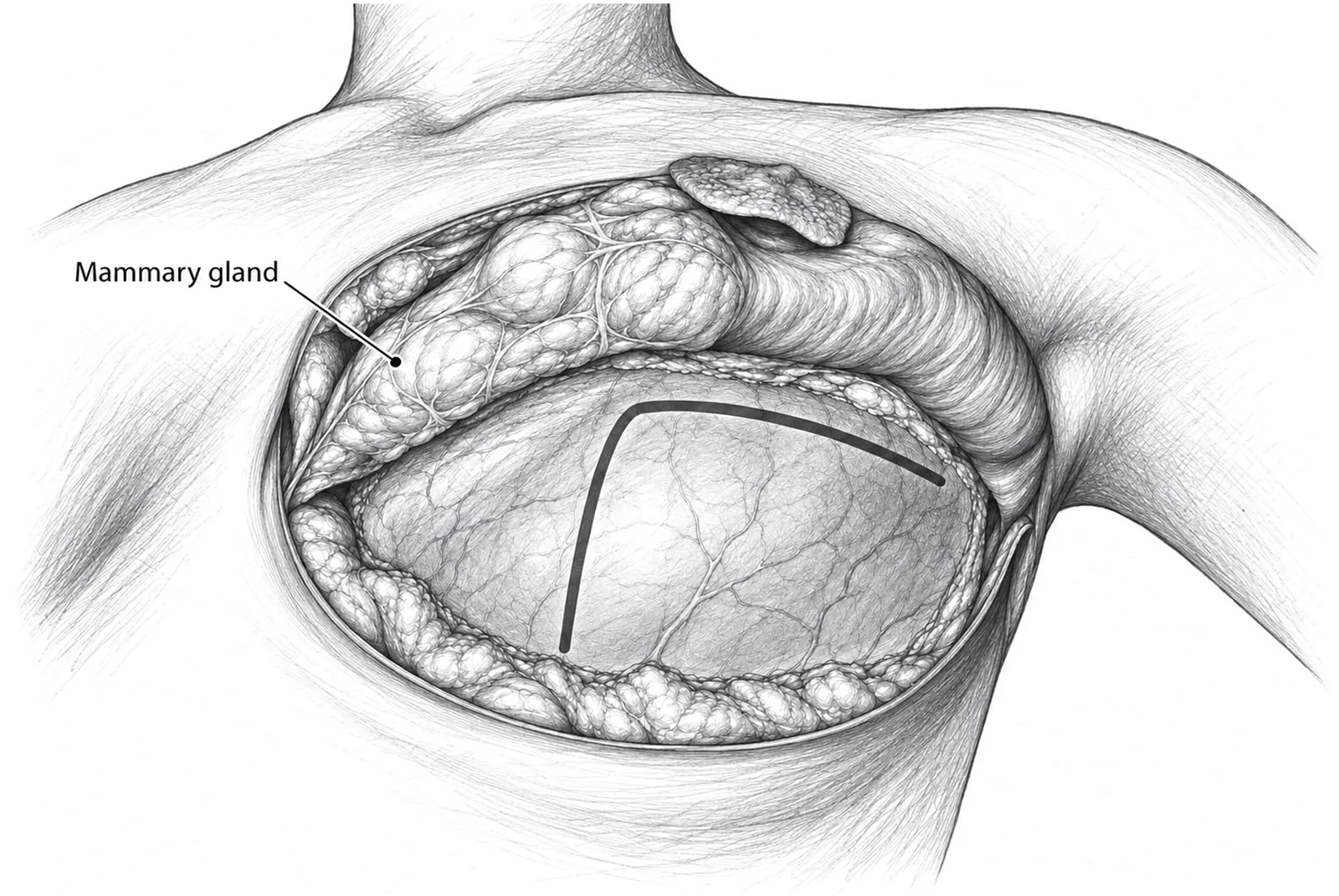
Capsule flap design.

**Fig. 5:**
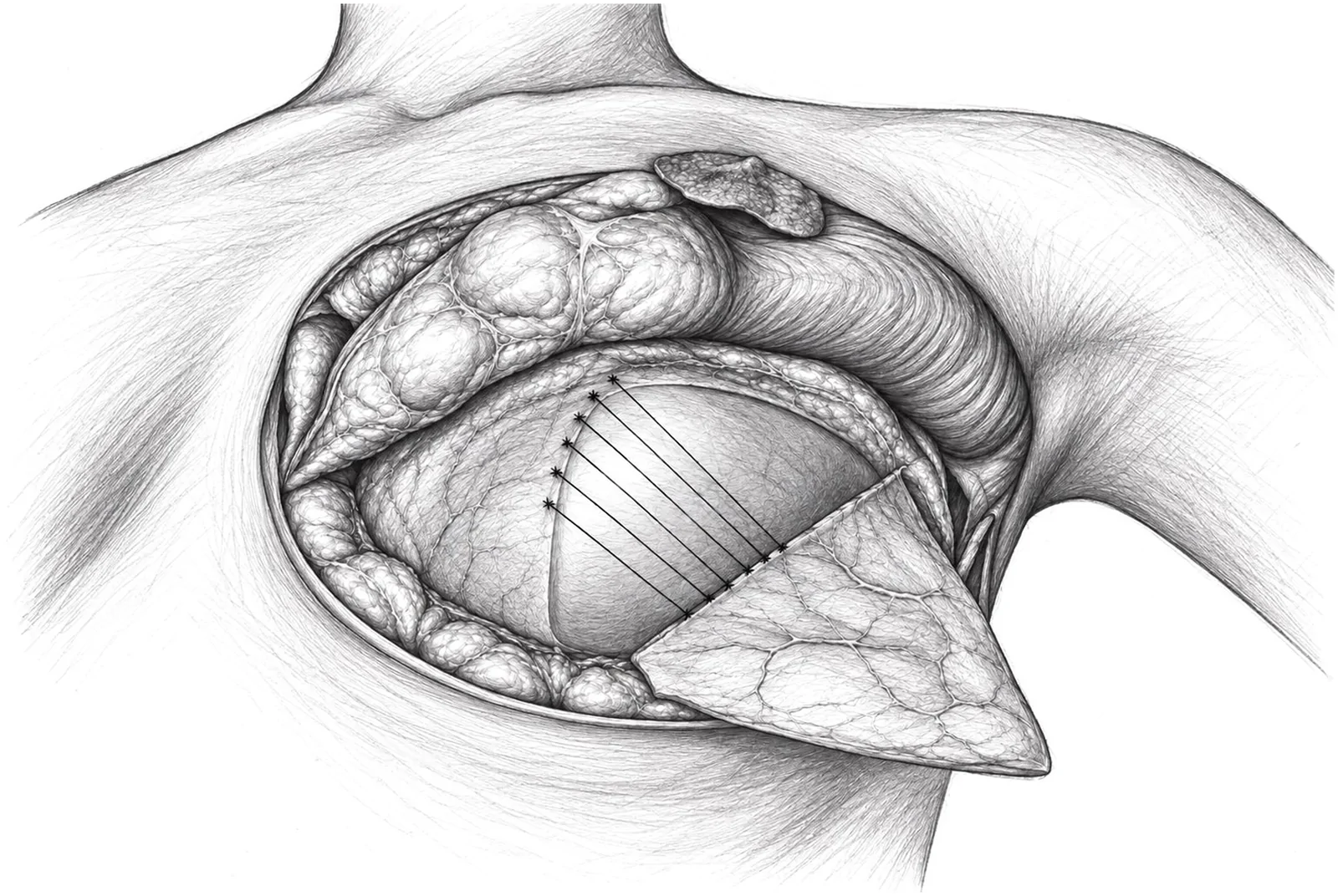
Flap advancement and first anchor line.

**Fig. 6:**
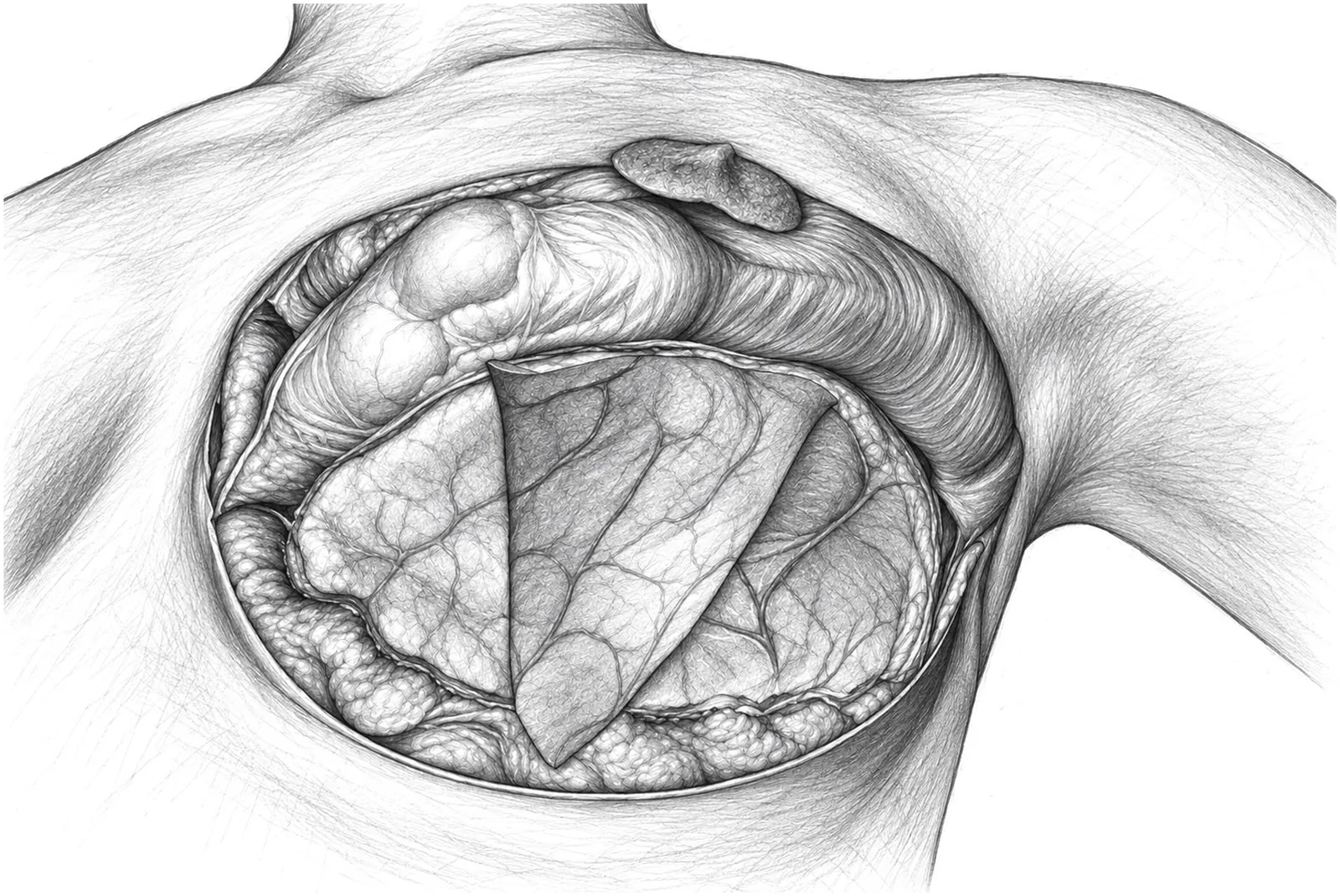
Flap remnant after first anchor line.

## Results

***Participants.*** All 30 eligible patients underwent the procedure and completed 12-month follow-up (Fig. 7). No patients were excluded or lost to follow-up.

**Fig. 7:**
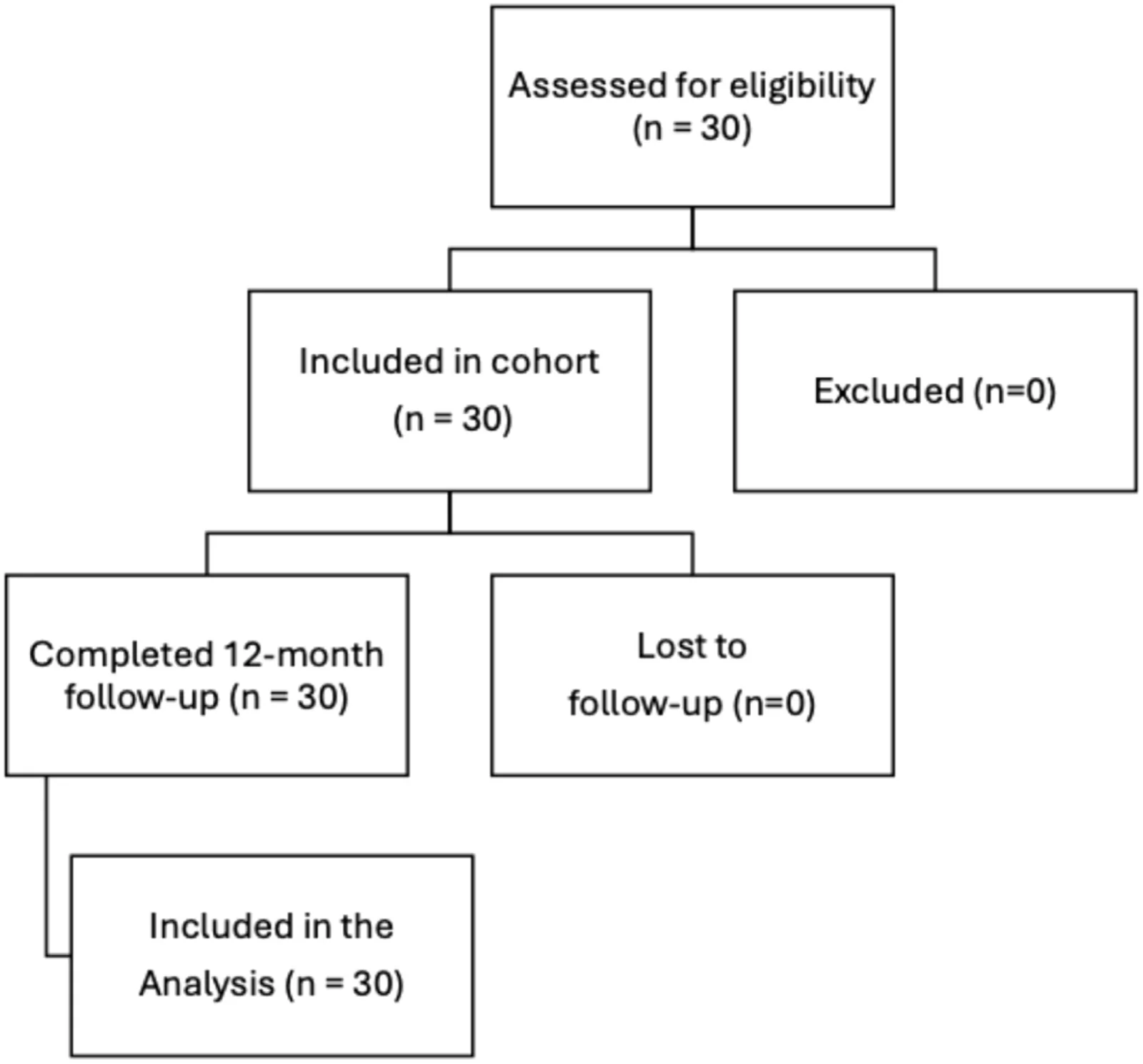
Study enrollment and follow-up of patients undergoing the Double-Anchor Capsule Flap Technique. Study enrollment.

Unadjusted outcome estimates were calculated descriptively for all patients included in the study. At one-year follow-up, flap survival was achieved in 100% of cases (30/30). The mean reduction in implant volume was 22.3 mL (SD 25.9), with a 95% confidence interval (CI) of 13.1–31.5 mL. No postoperative complications, recurrent ptosis, implant malposition, or flap loss were observed (0%, 95% CI 0–11.6%). In absolute terms, the risk of postoperative complications, recurrent ptosis, implant malposition, and flap loss over the 12-month follow-up period was 0%. Due to the single-group retrospective case series design, no confounder-adjusted analyses were performed. Potential confounders, including patient age, body mass index (BMI), implant size, implant age, implant plane, and prior surgical history, were recorded a priori and considered clinically relevant because of their known influence on implant stability and breast contour. These variables were evaluated descriptively to aid interpretation of outcomes rather than for statistical adjustment.

No subgroup, interaction, or sensitivity analyses were performed due to the single-group retrospective design and absence of a comparator group.

***Patient Demographics:*** Thirty patients underwent breast remodeling using the Double-Anchor Technique (DAT) during revision mastopexy. Mean BMI was 27.6. Patient demographics and baseline characteristics are described in Table 1.

**Table 1:** Baseline characteristics.

**Patient #**	**BMI**	**Age**	**Implant Age**	**Implant Size (Preoperative)**	**Implant Brand (Preoperative)**	**Implant Plane (Preoperative)**	**Baker Scale**
1	24	35	8	355	Sientra	Subglandular	I
2	28	42	10	430	Mentor	Submuscular	II
3	29	39	4	480	GC Aesthetics	Dual Plane	II
4	26	37	11	390	Allergan	Submuscular	II
5	32	51	12	570	BellaGel	Dual Plane	I
6	24	34	8	330	Polytech	Submuscular	I
7	30	45	13	525	Eurosilicone	Submuscular	II
8	29	43	10	400	Silimed	Submuscular	II
9	23	36	11	355	Silimed	Submuscular	II
10	27	44	5	460	GC Aesthetics	Submuscular	II
11	30	49	13	395	BellaGel	Subglandular	I
12	24	41	8	330	Silimed	Dual Plane	I
13	32	52	3	510	Motiva	Submuscular	II
14	23	33	4	395	Mentor	Submuscular	I
15	27	42	11	360	Natrelle	Submuscular	II
16	34	50	13	650	Motiva	Dual Plane	II
17	30	46	11	485	Sientra	Dual Plane	II
18	28	40	10	430	Allergan	Submuscular	I
19	22	31	9	500	Polytech	Subglandular	II
20	25	34	8	430	Eurosilicone	Dual Plane	II
21	27	36	10	570	BellaGel	Dual Plane	I
22	29	33	3	510	GC Aesthetics	Submuscular	II
23	29	34	9	580	Motiva	Dual Plane	II
24	26	32	7	400	Allergan	Submuscular	I
25	30	35	11	480	Mentor	Dual Plane	II
26	30	36	10	420	Polytech	Submuscular	II
27	28	33	9	530	Eurosilicone	Dual Plane	II
28	27	31	8	395	Silimed	Subglandular	II
29	30	38	12	435	Sientra	Subglandular	II
30	28	34	5	340	GC Aesthetics	Submuscular	II
Mean	27,6	38,9	8,9	448			
SD	2,9	6,2	3	82,5			
Range	22,1 - 34,0	31 - 52	3 - 13	330 - 650			

***Capsular Flap Viability:*** Flap survival was achieved in all 30 patients. A Jackson-Pratt™ (JP) drain was placed intraoperatively to manage postoperative serosanguineous fluid.

***Postoperative follow-up:*** Postoperative flap monitoring consisted of serial clinical examinations during hospitalization and outpatient follow-up visits. Evaluations included assessment of breast contour, localized firmness, signs of fat necrosis, wound healing, skin viability, temperature changes, erythema, seroma formation, and contour irregularities suggestive of vascular compromise. No cases of partial or complete flap loss were identified during the follow-up period. At the 1-year postoperative follow-up appointment, all patients demonstrated stable results with optimal nipple-areola complex position, breast shape, and projection. Post-surgical outcomes and one-year follow-up data are presented in Table 2. Pre and postoperative clinical images are shown in Figs. 8, 9, Fig. 10, Fig. 11.

**Table 2:** Post-surgical data.

**Patient #**	**Implant Plane (Postoperative)**	**Implant Reused**	**Implant Size (Postoperative)**	**Reduction (cc)**
1	Motiva	No	355	0
2	GC Aesthetics	No	390	40
3	GC Aesthetics	Yes	480	0
4	Motiva	No	380	10
5	Motiva	No	550	20
6	GC Aesthetics	No	330	0
7	Motiva	No	500	25
8	GC Aesthetics	No	400	0
9	Motiva	No	350	5
10	GC Aesthetics	Yes	460	0
11	GC Aesthetics	No	360	35
12	Motiva	No	300	30
13	Motiva	Yes	510	0
14	GC Aesthetics	No	360	35
15	Motiva	No	350	10
16	Motiva	No	550	100
17	GC Aesthetics	No	480	5
18	Motiva	No	400	30
19	GC Aesthetics	No	500	0
20	Motiva	No	400	30
21	GC Aesthetics	No	560	10
22	GC Aesthetics	Yes	510	0
23	Motiva	No	500	80
24	GC Aesthetics	No	360	20
25	Motiva	No	450	30
26	GC Aesthetics	No	390	30
27	Motiva	No	450	80
28	GC Aesthetics	No	360	35
29	Motiva	No	425	10
30	GC Aesthetics	Yes	340	0
Mean			425,0	22,3
SD			74,4	25,9
Range			300 - 560	0 - 100

**Fig. 8:**
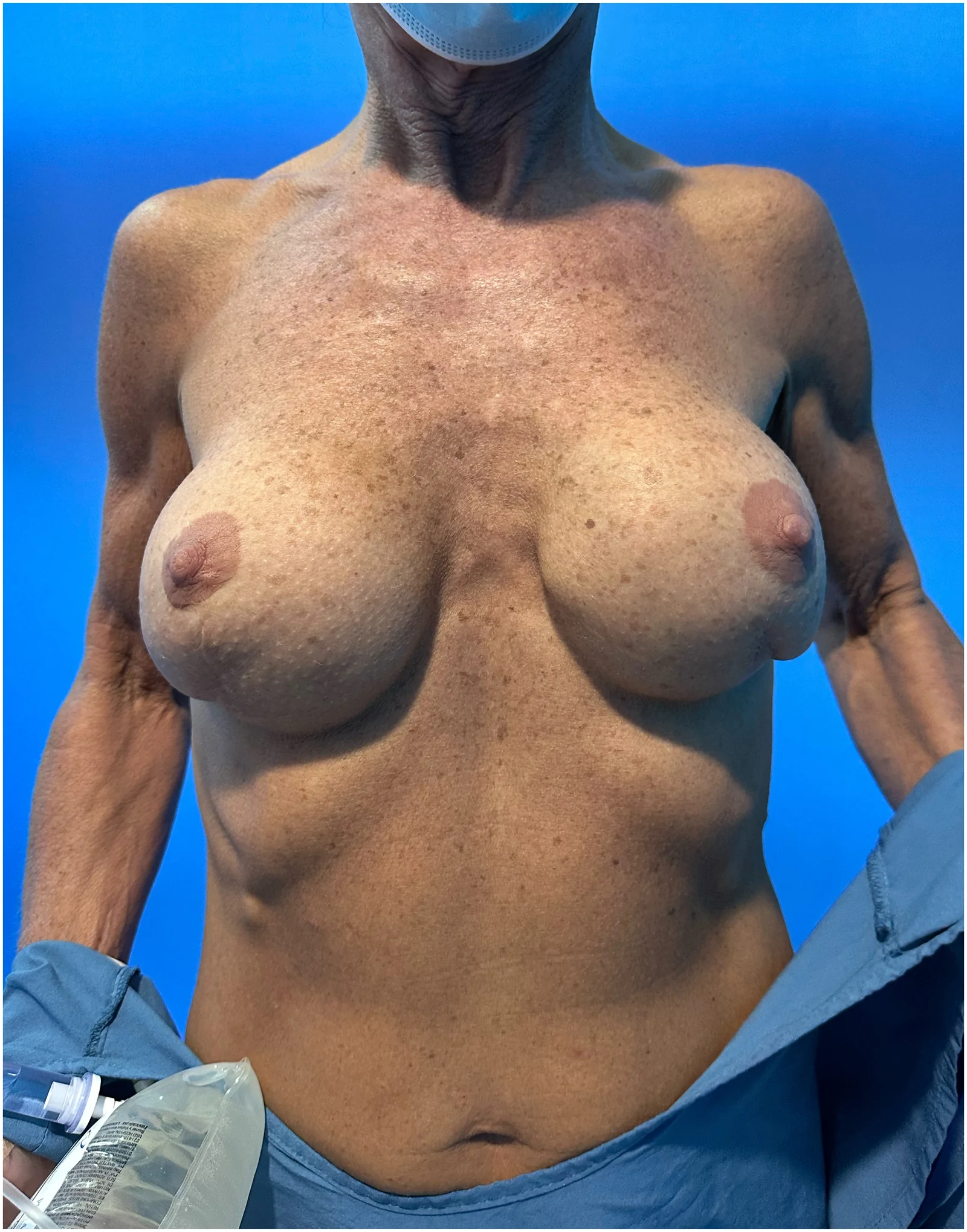
Clinical image. Preoperative - Front view. **Case 3: Description:** Secondary mastopexy with retractable scar corrected and stabilized with the Double Anchor Transposition Flap Technique. One year follow-up.

**Fig. 9:**
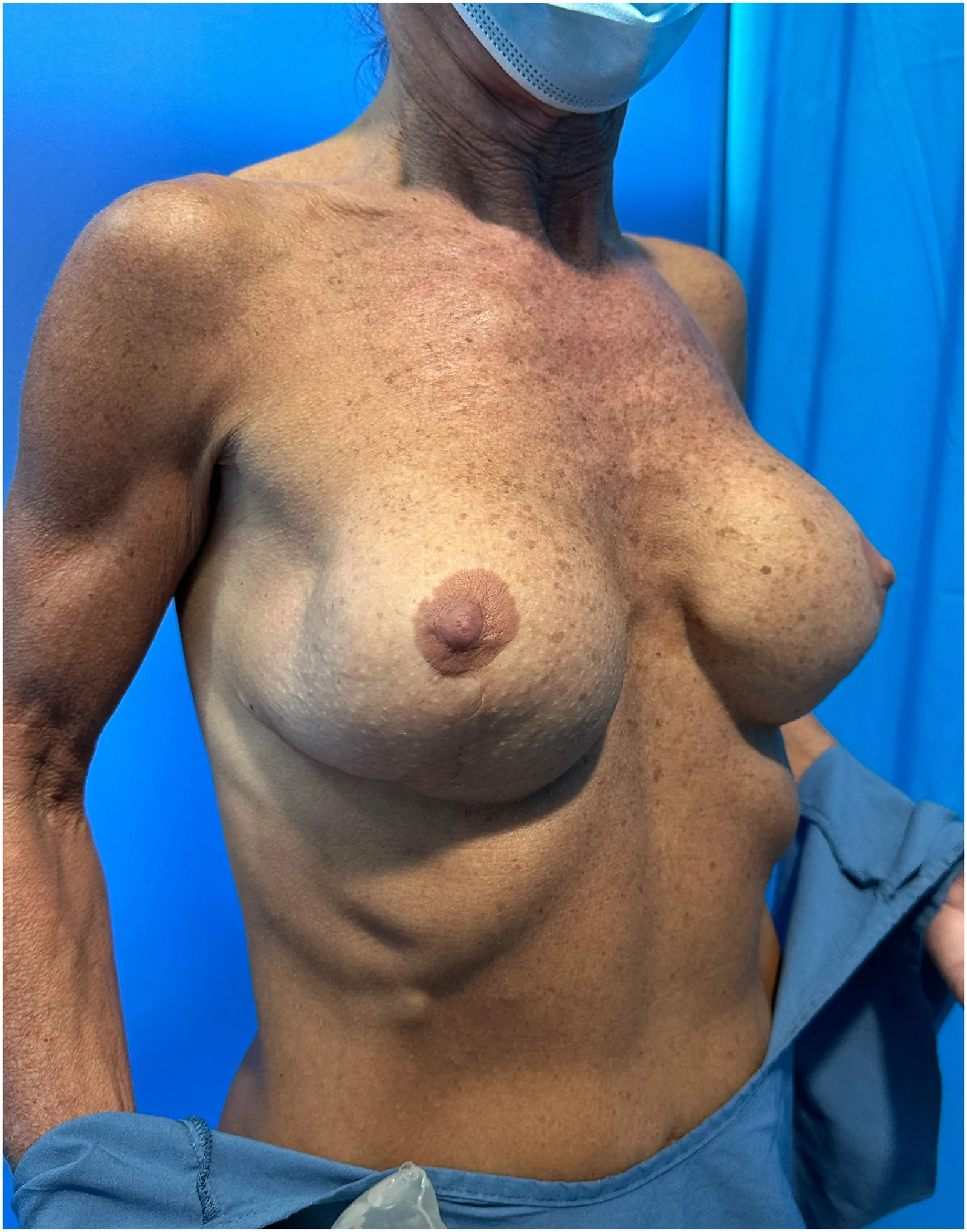
Clinical image. Preoperative - Right oblique view.

**Fig. 10:**
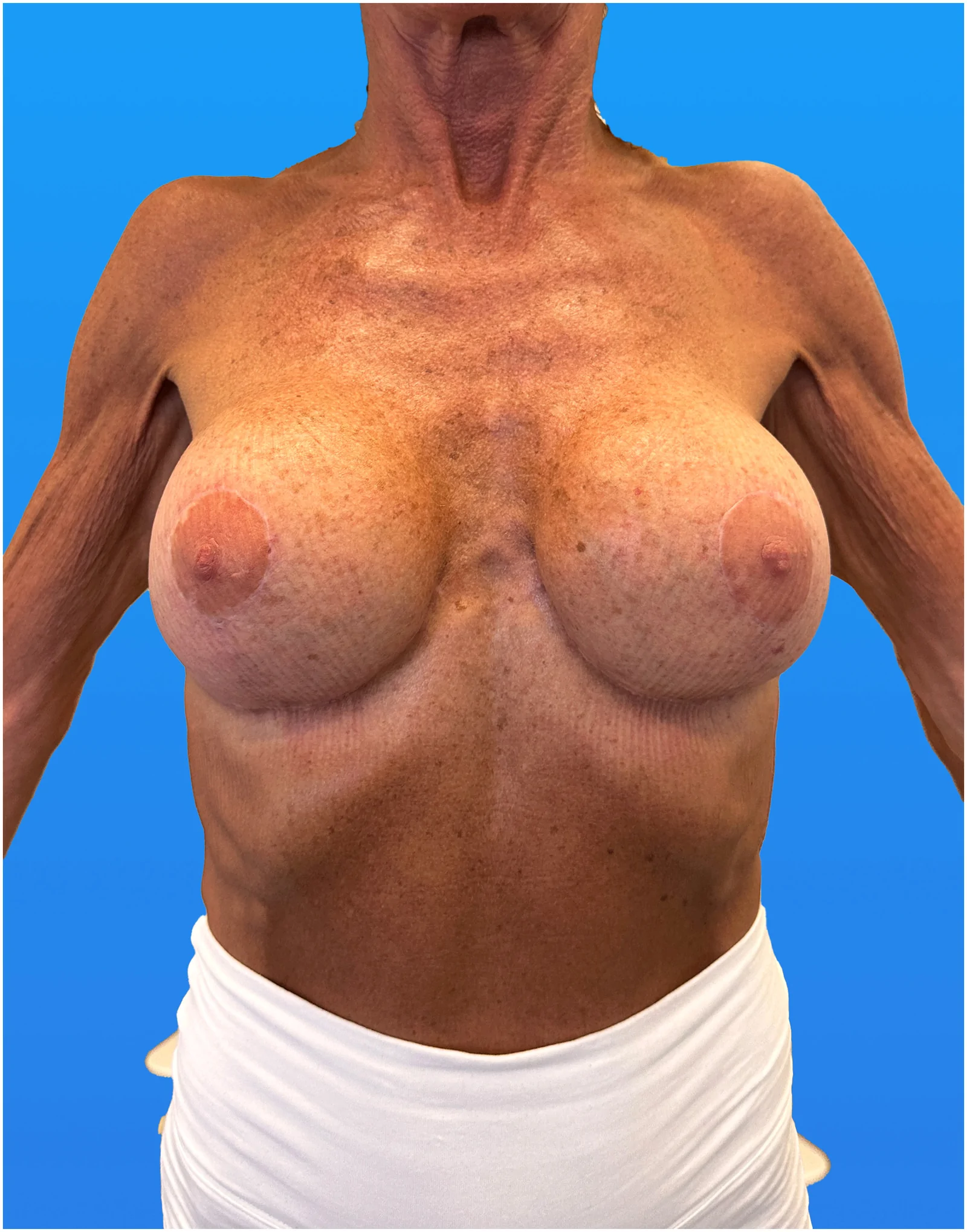
Clinical image. Postoperative - Front view.

**Fig. 11:**
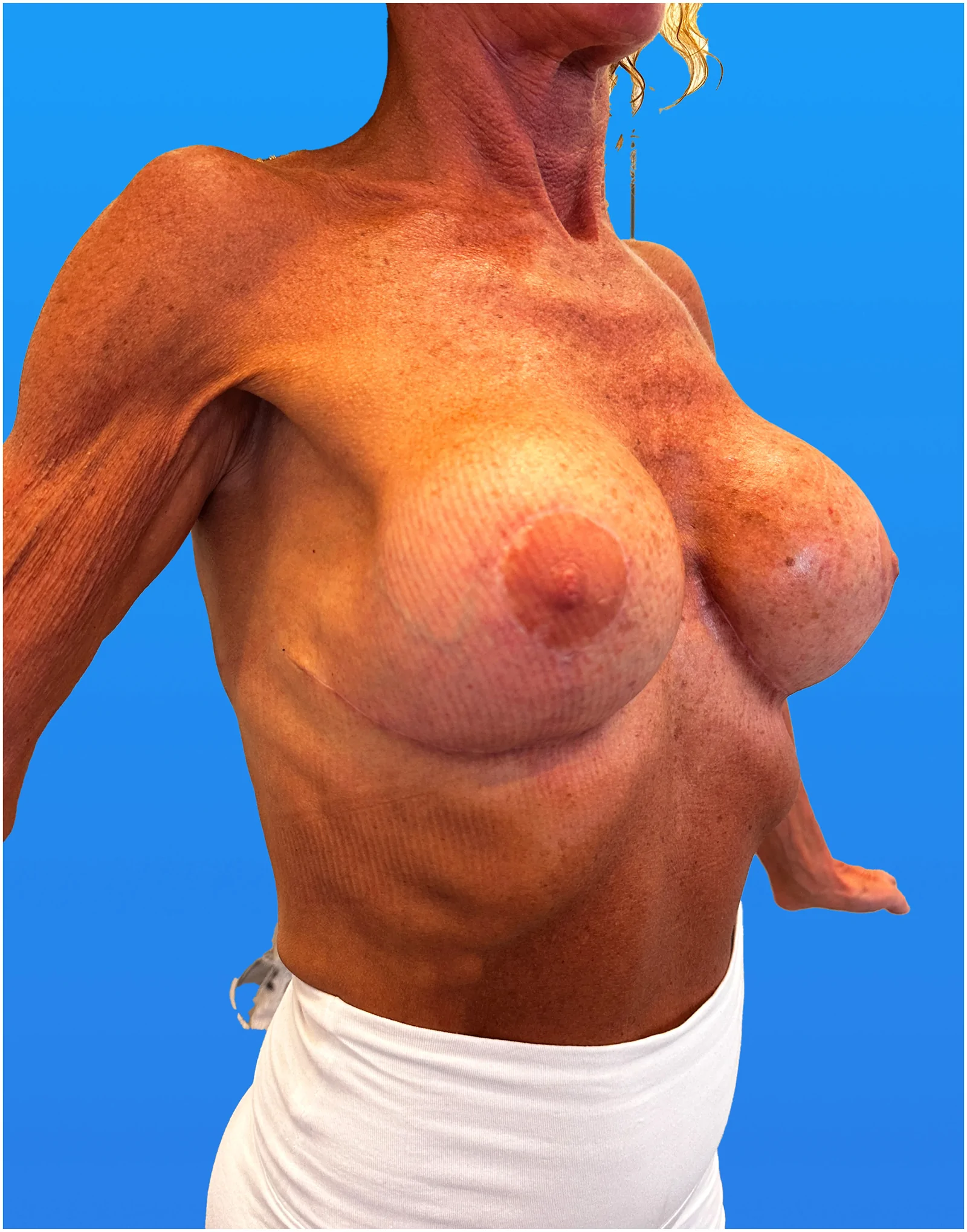
Clinical image. Postoperative - Right oblique view.

***Complications:*** Postoperative complications were documented from medical records up to one year. A complication was defined as any issue at the surgical site, including wound dehiscence, infection, seroma, fat necrosis, re-exploration, and flap loss. No complications at the incision intersection were observed and no recurrent ptosis or implant malposition was observed. Zero reports of recurrent ptosis or implant malposition were observed. No cases of BIA-ALCL were identified during follow-up; the study was not designed to assess its incidence.

## Discussion

The objective of this retrospective case series was to describe the Double-Anchor Capsule Flap Technique (DAT) and evaluate its safety and effectiveness for stabilizing breast implants during revision mastopexy.

***Key results:*** In this 30-patient case series, flap survival was 100%, with stable breast shape, optimal NAC positioning, and consistent implant projection at 1-year follow-up. No postoperative complications, recurrent ptosis, or implant malposition were observed. Mean implant volume reduction was 22.3 mL (SD 25.9), intended to reduce eccentric forces and improve implant stability. These findings support DAT’s safety and effectiveness in revision mastopexy.

Preservation of the physiological capsule may be an optimal strategy for prosthesis stabilization in distended implant pockets. Contributing factors include lateral muscular overdissection, compensatory hypertrophy in dual-plane techniques, lower pole distension in prepectoral pockets, and tissue atrophy combined with gravitational effects of large implants.

Indications for total capsulectomy include capsulitis, pathological encapsulation, calcification, or BIA-ALCL. In their absence, capsule preservation is advisable. Total capsulectomy during implant replacement may increase scarring, potentially exceeding baseline fibrosis due to bleeding-related stimulation.

Although no cases of BIA-ALCL were identified, the rarity of the disease, limited sample size, and follow-up preclude conclusions regarding risk reduction. This technique was developed to improve implant support and long-term breast contour, not for BIA-ALCL prevention or diagnosis. Ongoing vigilance remains essential.

The use of polyurethane-coated implants to promote adhesion and capsule integration carries risk, including reported associations with breast implant-associated anaplastic large cell lymphoma (BIA-ALCL), warranting caution. If capsule abnormalities, disease, or infiltration are suspected, histopathological analysis is recommended and capsule flap creation should be avoided.

Changing the prosthesis plane may reduce cephalocaudal forces from gravity and muscle activity. If upper pole coverage is adequate, an inverted dual-plane approach can allow the capsule and gland to provide superior support. Another possible strategy is weakening the muscle through myotomy, as described by Mario Pelle Ceravolo^10^ and Carlos Uebel et al.,^11^ to facilitate ascent and greater projection of the upper pole. Finally, if the implant is prepectoral, the use of a total submuscular plane could be considered.

Considering the impact of prosthesis volume on atrophy, descent, and malposition, implant volume reduction may minimize eccentric forces and gravitational effects. In this series, mean implant reduction was 22.3 mL, as reported in the statistical analysis. Most implants were not reused (25 patients), while 5 pairs were reused.

***Limitations:*** Retrospective case series lack control groups, limiting comparisons and causal inference. Small sample size and short follow-up reduce precision and may miss rare or delayed events. Reliance on records and photographic assessment may introduce information bias despite standardized data collection and uniform follow-up. These limitations are inherent to the study design and should be considered when interpreting results.

***Interpretation:*** This case series suggests that the Double-Anchor Capsule Flap Technique (DAT) provides reliable implant stabilization, dual support, and maintained breast contour at 1 year. Although outcomes were favorable, the small sample size, single-center design, lack of control group, and limited follow-up preclude definitive conclusions on rare complications or long-term durability. Comparisons with prior capsular flap and internal bra techniques suggest potential advantages in stability, but prospective studies are needed to confirm these findings and assess generalizability.

**Generalisability:** This case series reflects outcomes in adults with prior breast augmentation, BMI 20–35, and adequate flap thickness, treated by a single experienced surgeon at a high-volume center. Caution is warranted when extrapolating results to different patient populations or settings. Larger multicenter studies are needed to assess whether the benefits of the Double-Anchor Capsule Flap Technique (DAT) are generalizable.

## Conclusion

The Double-Anchor Capsule Flap Technique (DAT) offers a promising approach for correcting breast deformities after augmentation or mastopexy. This technique provides dual support via two anchor lines, enhancing long-term stability and reducing prosthesis displacement from gravitational and muscular forces. The study also highlights the role of implant volume reduction in minimizing eccentric forces that contribute to atrophy and malposition.

Careful patient selection and meticulous preoperative planning are crucial to the success of this technique. No BIA-ALCL cases were observed, but this finding should be interpreted in the context of the rarity of the disease and the scope of our study.

Overall, the Double-Anchor Capsule Flap Technique is a useful tool for plastic surgeons, offering an effective option for correcting complex breast deformities. Further research with larger cohorts and longer follow-up is needed to confirm long-term outcomes and refine the technique for broader use.

## Ethical approval

Not required.

## Consent

All patients provided informed consent for inclusion, publication of anonymized data, and clinical images.

## Funding

No external funding was received for this study. The authors had full responsibility for study design, data collection, analysis, interpretation, and writing of the manuscript.

## STROBE statement compliance

This study is reported in accordance with the STROBE guidelines for observational studies. The completed STROBE checklist has been submitted as supplementary material.

## Declaration of generative AI and AI-assisted technologies in the manuscript preparation process

During the preparation of this work the author(s) used Chat GPT in order to improve grammar and syntax. After using this tool/service, the author(s) reviewed and edited the content as needed and take(s) full responsibility for the content of the published article.

## Declaration of competing interest

The authors declare that they have no conflict of interest.

## Appendix Supplementary materials

Video 1: Flap Advancement and Anchorage. Flap Advancement and Anchorage. Once the flap is anchored on the first line of fixation, the redundant flap is mobilized to the midline to the second line of fixation.

Video 2. Dissection. With non-absorbable threaded sutures using mattress stitches on desired anchorage points guided to the angle so the stabilization of the implant is assessed.

Video 3. Closing the Flap. Advancing the capsule flap and anchoring to the midline after the position of the implant is estimated.

Video 4. Video animation. Surgical procedure. Three-Dimensional Procedure Overview.
